# Editorial: The Role of Protein Post-Translational Modifications in Protein-RNA Interactions and RNP Assemblies

**DOI:** 10.3389/fmolb.2021.831810

**Published:** 2022-01-04

**Authors:** Roberto Giambruno, Ewa A. Grzybowska, Nicolas L. Fawzi, Dorothee Dormann

**Affiliations:** ^1^ Center for Genomic Science, Italian Institute of Technology (IIT), Milano, Italy; ^2^ Maria Sklodowska-Curie National Research Institute of Oncology, Warsaw, Poland; ^3^ Brown University, Providence, RI, United States; ^4^ Biocenter, Johannes Gutenberg University Mainz, Mainz, Germany; ^5^ Institute of Molecular Biology (IMB), Mainz, Germany

**Keywords:** PTMs (post-translational modifications), RNA-protein interactions, RNP granules, phase separation, intrinsically disordered region (IDR)

RNA binding proteins (RBPs) are crucial regulators that participate in almost every cellular function by contributing to key biological processes such as transcription, translation, RNA splicing and RNA transport (Gerstberger et al., 2014). Posttranslational modifications (PTMs) control different aspects of RBPs, in particular their cellular localization; their stability and turnover; the ability of RBPs to bind to RNA and other proteins; and the propensity of RBPs to undergo liquid-liquid phase separation (LLPS). The most well characterized PTMs of RBPs are phosphorylation, methylation, acetylation, sumoylation and ubiquitinylation. The first four of these PTMs have been mainly linked to the regulation of RBPs cellular distribution and interactions, while ubiquitinylation is mainly involved in protein degradation and turnover (Sternburg et al., 2022). Recently, all of these PTMs have been described to regulate the LLPS behavior of RBPs and the consequent formation of membraneless-organelles (MLOs), such as stress granules (SGs) or other ribonucleoprotein (RNP) granules (Wiedner and Giudice, 2021).

The goal of our research topic is to highlight how PTMs regulate RNA-protein interactions, protein-protein interactions, LLPS of RBPs, and RNP granule formation and dynamics, as well as how altered PTM patterns on RBPs can be linked to human diseases.

The review by Velázquez-Cruz et al. summarizes the impact that PTMs have on several mammalian RBPs and how an aberrant PTM profile causes an alteration of physiological processes leading to diseases, such as cancer and neurodegenerative disorders. In particular, alterations of the PTM profile or mutations in post-translationally modified amino acids in RBPs like trans-activating response element DNA-binding protein of 43 kDa (TDP-43), fused in sarcoma (FUS) and heterogeneous nuclear ribonucleoprotein A and B type (hnRNP-A/B) are linked to neurodegenerative disorders, such as amyotrophic lateral sclerosis (ALS), as summarized by Farina et al. This aspect has also been covered in a review by Clarke et al. of biochemical and functional characterization of heterogeneous nuclear ribonucleoprotein A1 (hnRNPA1), belonging to the hnRNP-A/B subfamily, in both physiological conditions and neurodegenerative diseases; and how PTMs modulate hnRNPA1 molecular functions.

Among the numerous PTMs reported so far, arginine methylation, phosphorylation and sumoylation seem to play major roles in the regulation of RBP activities and in particular on their RNA-binding properties. This last aspect is extremely relevant but at the same time challenging because the current quantitative affinity purification processes, both protein- and RNA-centric strategies, are biased in the assessment of the interactions of a specific RBP and its respective targets, as reviewed by Vieira-Vieira and Selbach. To overcome this challenge, Maniaci et al., used an alternative quantitative proteomic approach that is based on orthogonal organic phase separation (OOPS) (Queiroz et al., 2019) to profile global effects on RNA-protein interaction dynamics exerted by the modulation of protein arginine methyltransferase (PRMT) activity, revealing differences caused by an altered arginine methylation pattern of RBPs. The role of PRMTs in RBP binding activities has also been reported in pathogenic kinetoplastids, as summarized by Campagnaro et al. The authors suggest that the activity of PRMTs can be pharmacologically inhibited paving the way to repurposing these drugs and the development of novel anti-parasite strategies.

Arginine methylation is also a key regulator of phase transition and RNP granules dynamics (Hofweber and Dormann, 2019). *In vitro* studies have demonstrated that arginine methylation often reduces RBP phase separation; however, in the germ line arginine methylation promotes the formation of RNP condensates, as reviewed by Schisa and Elaswad. Thus, there might be a discrepancy between *in vitro* and *in vivo* results that can be explained by the presence of additional factors or other PTMs (and hence a PTM crosstalk) in eukaryotic cells. Indeed, phosphorylation and arginine methylation can be juxtaposed on the same RBP and can have a synergistic or antagonistic effect in the regulation of RBP phase separation. Along these lines, Lenard et al. discovered that the serine-arginine protein kinase 1 (SRPK1) phosphorylates the cold-inducible RNA-binding protein (CIRBP) in the RG/RGG regions, thus impairing arginine methylation of RGG/RG motifs and suppressing CIRBP LLPS. At the same time, arginine methylation of CIRBP RG/RGG regions precludes any phosphorylation event by SRPK1. Hence, LLPS of CIRBP is co-regulated by both phosphorylation and methylation of the same CIRBP region.

Similarly, sumoylation is known to regulate LLPS of RBPs and in particular the formation of different MLOs, including P-bodies, nucleoli and stress granules, as reviewed by Keiten-Schmitz et al. In addition, the SUMO pathway has a crucial role in the disassembly of stress granules through the activation of the SUMO-targeted ubiquitin ligase (StUbl) pathway. As summarized by the authors, the StUbl pathway contrasts the formation of aberrant stress granules observed in neurodegenerative disorders, such as ALS, opening new perspectives for the understanding and cure of these diseases.

Overall, the articles collected in this Research Topic highlight the impact that PTMs have on RBP interactions and functions. In addition, they shed light on the crosstalk between different PTMs that finely regulate the generation and disassembly of RBP condensates. These aspects can be relevant for the future development of therapeutic strategies aimed at regulating aberrant LLPS of RBPs that are strongly linked to neurodegenerative disorders.
